# The Effect of Transcutaneous Electrotherapy on Lumbar Range of Motion and Paraspinal Muscle Characteristics in Chronic Low Back Pain Patients: A Systematic Review and Meta-Analysis

**DOI:** 10.3390/jcm12144680

**Published:** 2023-07-14

**Authors:** Daniel Wolfe, Brent Rosenstein, Maryse Fortin

**Affiliations:** 1 Department Health Kinesiology and Applied Physiology, Concordia University, Montreal, QC H4B 1R6, Canada; daniel.wolfe@mail.concordia.ca (D.W.); brent.rosenstein@icloud.com (B.R.); 2 PERFORM Centre, Concordia University, Montreal, QC H4B 1R6, Canada; 3 Centre de Recherche Interdisciplinaire en Réadaptation (CRIR), Montreal, QC H4B 1T3, Canada

**Keywords:** chronic low back pain, low back pain, musculoskeletal pain, rehabilitation, TENS, NMES, IFC, EMS

## Abstract

Chronic low back pain (CLBP) affects paraspinal muscle size, quality (e.g., fatty infiltration), range of motion (ROM), and strength. Although transcutaneous electrotherapies are used to treat CLBP, their effects on paraspinal-related outcomes are not fully known. The aim of this systematic review and meta-analysis was to assess the overall effect of transcutaneous electrotherapies on trunk/lumbar ROM, paraspinal muscle morphology, and trunk muscle function (including strength and endurance) in CLBP patients. A systematic search of four databases and two study registers was conducted between 1 February 2022 and 15 September 2022. Two reviewers were responsible for screening and data extraction. Of the 3939 independent records screened, 10 were included in the systematic review and 2 in the meta-analysis. The results suggest there is limited evidence that both EMS and EMS *plus* exercise are superior to passive and active controls, respectively, for improving trunk muscle endurance. There is limited evidence that neither TENS nor mixed TENS are superior to controls for improving trunk muscle endurance. There is limited evidence that NMES is superior to passive controls for improving trunk muscle strength. The effect of transcutaneous electrotherapy on the other investigated outcomes was inconclusive. Future transcutaneous electrotherapy studies should focus on paraspinal-based outcomes that are under-studied.

## 1. Introduction

Chronic low back pain (CLBP) affects approximately 20% of the global population [1] and is the leading cause of years lived with disability worldwide [2], in spite of large healthcare expenditures towards its treatment [3]. There is growing evidence of morphological and functional changes to the paraspinal muscles in the presence of CLBP, such as reduced muscle size [4] and activation [5] and increased fat infiltration [6]. Changes to paraspinal muscle strength and endurance may also occur in conjunction with CLBP [7]. However, guidelines for the development of systematic reviews for CLBP have not highlighted the importance of evaluating objective changes to paraspinal muscle composition and function nor have they traditionally recommended examining fear-avoidance beliefs and pain catastrophizing as part of patient-centered outcomes [8,9].

Conservative treatments for CLBP include manual therapy, exercise, pharmacological interventions, and transcutaneous electrotherapy, among others. Transcutaneous electric nerve stimulation (TENS) is an electrotherapy that aims to stimulate sensory nerve fibres and is thought to promote analgesia by activating endogenous inhibitory pathways in the central nervous system and by reducing peripheral nociceptive output [10]. TENS continues to be used in the treatment of CLBP in spite of a lack of clear endorsement by medical guidelines [11,12]. The Cost B13 Working Group on Guidelines for Chronic Low Back Pain does not recommend TENS for CLBP, citing ‘strong evidence’ that it is not more effective than placebo or sham TENS [11], while the Low Back Pain Working Group of the North American Spine Society’s Evidence-Based Clinical Guideline Development Committee found conflicting evidence that TENS improves pain or function in the short–medium term [12]. However, recent systematic reviews [13,14] evaluating the effect of TENS on CLBP, with a focus on intervention timing and outcome assessment, have found a modest but positive effect on pain and function under certain conditions. For example, Jauregui et al. (2016) found significant weighted mean differences in pain intensity from pre- to post-treatment in patients treated with TENS for less than, but not more than, 5 weeks [13]. Wu et al. (2018) found that TENS improves functional disability when follow-up is within 6 weeks of treatment, compared to controls [14]. One limitation of these reviews is that their inclusion criteria were broad: for example, Wu et al. (2018) [14] included studies investigating both CLBP and LBP more generally. Another limitation is that neither examined paraspinal muscle-related outcomes, which to our knowledge, have never been included in a systematic review of TENS for CLBP.

Other forms of transcutaneous electrotherapy used to treat CLBP include interferential current (IFC) and electromyostimulation (EMS). IFC is created when two medium frequency alternating currents are crossed to create a ‘low frequency’ resultant current. It is claimed that IFC produces lower skin impedance, allowing current to penetrate tissue more deeply than conventional TENS [15]. IFC is most used for pain relief but may be used for muscle therapy. Electromyostimulation (EMS) aims to stimulate motor fibres to produce involuntary muscle contraction and is used to improve activation and strength in weakened but innervated muscles [15]. Neuromuscular electrical stimulation (NMES), sometimes called electrical stimulation (ES), is a form of EMS generally delivered to muscle under static conditions to evoke visible muscle contractions. Functional electrical stimulation (FES) pairs muscle stimulation with a functional task such as grasping an object [16], while whole-body electromyostimulation (WB-EMS) involves performing resistance training in conjunction with muscle stimulation [17]. Until recently, no systematic review had evaluated the effect of EMS on CLBP outcomes. The Philadelphia Panel (2001) was unable to find eligible studies investigating the effect of EMS in subjects with CLBP [18]. Poitras and Brosseau (2008) attempted to assess the effect of EMS in CLBP as part of a larger review article but found no relevant studies [19]. Recently, Linzmeye et al. (2022) reviewed the effect of NMES on lumbopelvic muscle function and paraspinal muscle thickness in CLBP patients. They reported that NMES helps improve paraspinal muscle endurance, while the effects on paraspinal muscle thickness were mixed [20]. While this was the first systematic review to specifically investigate the effects of transcutaneous electrotherapy on paraspinal muscle morphology, (*i*) it was limited to NMES-like modalities, (*ii*) did not assess other measurable markers of paraspinal muscle function (such as ROM), and (*iii*) both stand-alone and mixed interventions were compared/included in the same analysis. 

To date, no study has comprehensively examined the effect of both sensory and motor transcutaneous electrotherapy on a wide array of observer-measured CLBP outcomes. Therefore, the aim of this systematic review was to assess the overall effect of transcutaneous electrotherapies on the following outcomes: trunk/lumbar ROM, paraspinal muscle morphology, and trunk muscle function (including strength and endurance) in patients with CLBP. The effect of transcutaneous electrotherapies was compared with passive (sham TENS, placebo) and active (other modalities) controls. Separate analyses were conducted for isolated electrotherapy interventions and mixed interventions. Focus was paid to short (post-intervention), medium (1 month post-intervention), and long-term (>1 month post-intervention) outcomes.

## 2. Materials and Methods

The current review protocol was registered with PROSPERO (ID number: CRD42023383928). This systematic review followed the recommendations suggested by the Preferred Reporting Items for Systematic reviews and Meta-Analyses (PRISMA) 2020 explanation and elaboration guidebook [21]. We also adhered to the guidelines proposed by Cochrane Neck and Back [22] for systematic reviews. The conceptualization and design of this systematic review was led by DW and supported by MF. Data extraction and the risk-of-bias assessment were conducted jointly by DW and BR. Data analysis and writing was performed by DW, and all three authors participated in the review and editing process. 

### 2.1. Selection Criteria

#### 2.1.1. Types of Studies

We included randomized and quasi-randomized control trials assessing the effect of transcutaneous electrotherapy in CLBP patients in comparison with a passive or active control (see below). In line with the Philadelphia Panel’s consensus opinion [18], only studies with ≥5 participants per treatment group were included. To adequately assess the effect of interventions over time, we only included studies with ≥8 treatments per group. Only English- or French-language articles were included.

#### 2.1.2. Participants

Participants aged 18–70 with a diagnosis of CLBP, defined as persistent pain between the lower ribs and gluteal fold, with or without leg pain, of at least 12 weeks duration were included in this review. Participants diagnosed with a specific spinal pathology, defined as one of the following: infection, tumour, previous lumbar surgery, osteoporosis, fracture, structural deformity (ex. scoliosis), inflammatory disorder (ex. ankylosis spondylosis), or cauda equina syndrome, were excluded. However, participants with lumbar disc herniation were included—provided they did not present with radicular symptoms—in line with evidence that disc degeneration and annulus tears (visible on T2-weighted MRI) are not necessarily painful [23]. Studies that included participants with mixed lower and upper back pain were excluded. Additionally, studies that included a mix of acute (<4 weeks) and chronic LBP patients as well as sub-acute (4–12 weeks) and chronic LBP patients were excluded, in line with suggestions that these conditions be considered separately [24].

#### 2.1.3. Types of Interventions

We included studies that use transcutaneous electrotherapy as the primary intervention for CLBP. In cases of studies using transcutaneous electrotherapy *plus* another intervention, transcutaneous electrotherapy had to account for at least 40% of the treatment program. This cut-off value was previously used by Macedo et al. (2009) in their systematic review of motor control exercises for persistent LBP [24]. Studies that compared the effect of transcutaneous electrotherapy against a passive or active control were included. Passive controls were defined as the following: sham electrotherapy (defined as having the device modified so that no current passes to skin-surface electrodes), usual care, and/or no treatment. Active controls were defined as the following: any non-transcutaneous electrotherapeutic intervention for CLBP, such as ultrasound, hot/cold packs, exercise, mobilization/manipulation, massage/soft tissue therapy, acupuncture, and non-transcutaneous electrotherapy. Studies comparing two transcutaneous electrotherapeutic treatments were be excluded, as were studies comparing the effect of transcutaneous electrotherapy *plus* another intervention against a third intervention (ex. TENS + hot pack vs. ultrasound), in line with recommendations by the Cochrane Back and Neck Group [22]. Additionally, studies where the method of determining stimulation intensity is according to manufacturers’ specifications, or where it is not described, were excluded. 

#### 2.1.4. Types of Outcome Measures

We included studies that assess at least one of the following outcomes: lumbar or trunk range of motion, paraspinal muscle morphology (muscle cross-sectional area, fat infiltration, thickness, stiffness), or paraspinal muscle function (muscle contraction, strength, or endurance). The effect of the interventions in the short term (post-intervention), medium (1 month post-intervention), and long term (>1 month post-intervention) were considered.

### 2.2. Search Strategy

The following bibliographic databases were searched for studies pertaining to CLBP and transcutaneous electrotherapy: PubMed, Scopus, Web of Science, and Embase. Additionally, the follow study registers were searched for protocols of the included studies: WHO International Clinical Trials Registry Platform (http://who.int/ictrp/en/, accessed on 15 September 2022) and the US National Institute of Health (https://clinicaltrials.gov, accessed on 15 September 2022). A search strategy was developed based on a literature review and with help from a reference librarian at Concordia University affiliated with the department of Health, Kinesiology, and Applied Physiology. Mesh terms and key words related to (1) low back pain, (2) electrical stimulation therapy, (3) TENS, and (4) NMES were used. The search strategy for PubMed is available in Appendix A. The initial search was performed between 1 February 2022 and 31 March 2022, and the database was updated for the last time on 15 September 2022. No time limit was applied to publication dates. Search results were compiled in the reference management software Zotero (version 5.0.96.3). 

### 2.3. Study Selection

Two reviewers (DW, BR) initially screened the search results for potential studies based on the study title and, where reasonable, the abstract. After excluding articles during this first round, the full text of the remaining articles was read by the same reviewers, and a global yes/no decision was made for each potential study based on the inclusion criteria identified in Section 2.1. In the case of disagreement over the inclusion of an article, a third reviewer (MF) was consulted, and a consensus decision between the three reviewers was taken. Study screening was managed using SR Accelerator (https://sr-accelerator.com, accessed on 15 September 2022).

### 2.4. Data Extraction and Risk of Bias 

Two reviewers (DW, BR) independently extracted data from each included study using a modified version of the extraction template developed by Cochrane Back and Neck [22]. Participant characteristics, interventions, comparisons, outcomes, analysis approach, results, and study sponsorship were recorded. 

Risk of bias was assessed using the revised Cochrane risk-of-bias tool (RoB 2) [25] for randomized trials. This tool examines five domains: randomization bias, bias due to deviations from intended interventions, missing outcome data bias, measurement bias, and bias in selection of the reported result. Bias is assessed on a per-outcome basis, and the tool uses signaling questions and an algorithm to guide reviewers to judgment. Each outcome is rated as follows: low risk, some concerns, and high risk. Both reviewers (DW, BR) strictly followed the instructions outlined in the full guidance document for the RoB 2 tool. In case of disagreement, a third reviewer (MF) was consulted. 

### 2.5. Statistical Analysis

Studies were grouped according to intervention vs comparator (active or passive), at short-, medium-, and long-term follow-up (subgroup analysis). Meta-analyses were conducted, using a random-effects model, when comparisons within a group were sufficiently homogenous with respect to PICO variables (population, intervention, comparator, outcome). A minimum of three comparisons were needed for a comparison to be eligible for meta-analysis; in such cases, the overall treatment effect of the intervention, with 95% confidence intervals, was calculated for each outcome. For continuous variables measured using different scales, the standardized mean difference (SMD) was calculated. Statistical heterogeneity was conducted using the Q-test and reported as the *I*^2^ statistic. We interpreted the statistic as follows: <40% suggests a low risk of heterogeneity, 40–75% a moderate heterogeneity, and >75% a high risk of heterogeneity [22]. We used the Review Manager statistical software (RevMan version 5.4.1) to conduct the meta-analyses. 

## 3. Results

### 3.1. Search Results

**Figure 1:**
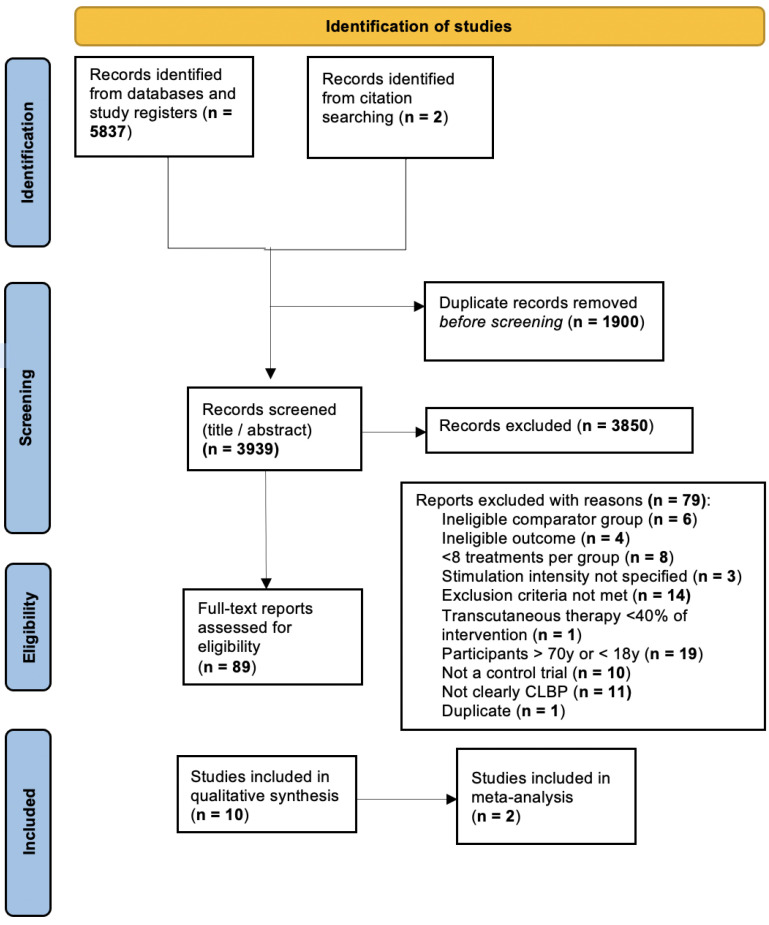
Study Flow Diagram.

We performed an electronic search for eligible articles across the following four databases: PubMed, Embase, Scopus, and Web of Science. We also hand searched reference lists for articles that the electronic search might have missed. A total of 5839 records were found through the search. After removing duplicates, 3938 titles were screened and 89 were selected for a full-text review. Ten studies were included in the qualitative review and two studies (spanning three comparisons) were eligible for meta-analysis. The full results of our search are presented below (Figure 1).

### 3.2. Risk of Bias

The risk of bias was evaluated on a per-outcome basis and ranged from ‘some concerns’ to ‘high’. Specifically, *bias in selected of the reported results* was judged to be of at least ‘some concerns’ for all outcomes because no protocols for statistical analysis could be found for any of the included studies; therefore, a comparison between the published report and the protocol could not be performed, which automatically elevates the risk of bias for this domain. In total, there were ‘some concerns’ of bias for 16 comparisons [17,26,27,28,29,30,31] and a high risk of bias for 4 comparisons [32,33,34]. The risk-of-bias assessment is presented in Supplementary Table S1. 

### 3.3. Characteristics of Included Studies

Of the ten included studies, eight were stand-alone [26,27,28,29,30,32,33,34] and two formed a pair with a shared recruitment process and inclusion/exclusion criteria but different interventions [17,31]. Four studies used sensory electrotherapy: three used TENS [28,33,34] and one used IFC [29]. The remaining six studies evaluated EMS: one used NMES [26], one used Russian current [32], one used Aussie current [30], one used mid-frequency (2500 Hz) current with progressive low-frequency (LF) modulation [27], and two used WB-EMS [17,31]. Furthermore, five studies comprised stand-alone transcutaneous electrotherapy interventions [27,28,29,30,32], four studies included only mixed interventions [17,26,31,33] (electrotherapy *plus* an additional intervention), and two studies evaluated both stand-alone and mixed interventions [27,28]. The study characteristics are provided in Supplementary Table S2, while the results are presented, per outcome, in Supplementary Tables S3–S5.

### 3.4. Outcome: Trunk/Lumbar ROM

#### 3.4.1. TENS versus Active Controls at Post-Intervention

One study assessed the effect of TENS against active control on trunk ROM in flexion and extension. Kofotolis et al. (2008) [28] compared 40–45 min of TENS with 30–45 min of rhythmic stabilization exercises. At post-intervention, the exercise group had significantly greater trunk flexion ROM than the TENS group (66.4 (5.4) vs. 61.1 (3.9), *p* < 0.05) and significantly greater trunk extension ROM than the TENS group (27.4 (1.1) vs. 24.5 (2.7), *p* < 0.05).

#### 3.4.2. TENS versus Active Controls at ≥1 Month Post-Intervention

Kofotolis et al. (2008) [28] re-assessed trunk ROM measurements at 1 and 2 months post-intervention. At 1 month post-intervention, the exercise group continued to have significantly greater trunk flexion ROM than the TENS group (73.8 (8.2) vs. 62.5 (15.3), *p* < 0.05) and significantly greater trunk extension ROM (28.9 (1.7) vs. 24.5 (2.9), *p* < 0.05). At 2 months post-intervention, the pattern held: the exercise group had greater trunk flexion ROM than the TENS group (75.7 (4.2) vs. 62.7 (1.5), *p* < 0.05) and greater trunk extension ROM (29.2 (2.1) vs. 24.9 (3.0), *p* < 0.05).

#### 3.4.3. TENS versus Passive Controls at Post-Intervention

Kofotolis et al. (2008) [28] compared 40–45 min of TENS with 40–45 min of sham TENS. At post-intervention, there were no significant differences in either trunk flexion or trunk extension ROM between the active and sham TENS groups.

#### 3.4.4. TENS versus Passive Controls at ≥1 Month Post-Intervention

Kofotolis et al. (2008) [28] re-assessed trunk ROM measurements at 1 and 2 months post-intervention. There were no significant differences in either trunk flexion or trunk extension ROM between the active and sham TENS groups at both time points.

#### 3.4.5. Mixed TENS versus Active Controls at Post-Intervention

Two studies [28,34] compared the effect of TENS *plus* exercise with exercise alone on spinal/lumbar ROM. Elserty et al. (2016) [34] assessed the effects of fixed TENS + 40 min of exercise, adjusted TENS + 40 min of exercise, and 40 min of exercise alone on spinal ROM in flexion and extension. With fixed TENS, the current amplitude remained constant throughout an entire treatment. With adjusted TENS, participants were asked at 5 min intervals whether the current strength had faded; if so, the amplitude was adjusted so that it returned to baseline levels. Kofotolis et al. (2008) [28] compared the effects of rhythmic stabilization exercises + TENS with rhythmic stabilization exercises alone on trunk ROM in flexion and extension. At post-intervention, Elserty et al. (2016) [34] reported that both the fixed and adjusted TENS groups had significantly greater spinal flexion ROM (50.3 (5.67), 51.0 (5.0)) than the exercise group (43.1 (4.66), *p* = 0.0001). Both mixed TENS groups also had significantly greater spinal extension ROM (19.27 (3.28), 19.73 (2.4)) than the exercise group (17.07 (2.49), *p* = 0.026). In contrast, Kofotolis et al. (2008) found that the rhythmic stabilization group had significantly greater trunk ROM in flexion than the mixed TENS group (66.4 (5.4) vs. 61.3 (3.2), *p* < 0.05). There were no significant between-group differences in trunk extension ROM. 

#### 3.4.6. Mixed TENS versus Active Controls at ≥1 Month Post-Intervention

Kofotolis et al. (2008) [28] re-assessed the effect of TENS + rhythmic stabilization exercises and rhythmic stabilization exercises alone at both 1 and 2 months post-intervention. At 1 month post-intervention, the exercise group had significantly greater flexion ROM than the mixed TENS group (73.8 (8.2) vs. 61.7 (15.8), *p* < 0.05) and significantly greater extension ROM than the mixed TENS group (28.9 (1.7) vs. 25.5 (4.0), *p* < 0.05). At 2 months post-intervention, the exercise group had significantly greater flexion ROM than the mixed TENS group (75.7 (10.2) vs. 63.7 (3.5), *p* < 0.05) and significantly greater extension ROM compared with the mixed TENS group (29.2 (2.1) vs. (26.0 (3.6), *p* < 0.05).

#### 3.4.7. Mixed TENS versus Passive Controls at Post-Intervention

Kofotolis et al. (2008) [28] compared TENS + rhythmic stabilization exercises with sham TENS. At post-intervention, there were no significant between-group differences in either trunk flexion or extension ROM. 

#### 3.4.8. Mixed TENS versus Passive Controls at ≥1 Month Post-Intervention

Kofotolis et al. (2008) [28] re-assessed the effect of TENS + rhythmic stabilization exercises and sham TENS 1 and 2 months post-intervention. At 1 month post-intervention, the mixed TENS group had significantly more trunk extension ROM than the sham TENS group (25.4 (4.0) vs. 22.0 (4.6), *p* < 0.05). There were no significant between-group differences in spinal flexion. At 2 months post-intervention, the mixed TENS group continued to have significantly more trunk extension ROM than the sham TENS group (26.0 (3.6) vs. 23.0 (4.1), *p* < 0.05). There were no significant between-group differences in spinal flexion.

#### 3.4.9. IFC versus Active Controls at Post-Intervention

Lara-Palomo et al. (2013) [29] assessed the effects of IFC with superficial massage on trunk flexion ROM. At post-intervention, there was no significant difference between the IFC and control groups in flexion ROM (MD = −1.12, 95% CI = −3.79, −1.54, *p* = 0.062).

### 3.5. Outcome: Trunk Muscle Strength

#### 3.5.1. Mixed EMS versus Active Controls at Post-Intervention

Both Alrwaily et al. (2019) [26] and Weissenfels et al. (2019) [31] assessed the effect of EMS on isometric trunk muscle strength. Alrwaily et al. (2019) reported no significant difference in isometric trunk *extension* strength between the mixed EMS and stabilization exercise groups. Weissenfels et al. (2019) reported no significant difference in the change in isometric *flexion* and *extension* strength, from baseline to post-intervention, between the WB-EMS and conventional exercise groups. 

#### 3.5.2. Mixed EMS versus Passive Controls at Post-Intervention

In their comparison of WB-EMS with passive control, Weissenfels et al. (2018) [17] reported that WB-EMS was significantly more effective at improving isometric trunk *extension* strength (MD = 8.29, 95% CI = 0.9, 16.4), but not isometric trunk *flexion* strength, at post-intervention.

### 3.6. Outcome: Trunk Muscle Endurance

#### 3.6.1. TENS versus Active Controls at Post-Intervention

Kofotolis et al. (2008) [28] compared the effect of rhythmic stabilization exercises with TENS for static and dynamic trunk muscle endurance in flexion and extension. Rhythmic stabilization exercises were significantly more effective than TENS at improving static flexion endurance (71.4 (4.2) vs. 59.2 (5.5), *p* < 0.05), static extension endurance (137 (6.9) vs. 82.5 (6.2), *p* < 0.05), dynamic flexion endurance (12.1 (1.6) vs. 7.5 (0.8), *p* < 0.05), and dynamic extension endurance (11.4 (1.5) vs. 8.8 (1.3), *p* < 0.05). 

#### 3.6.2. TENS versus Active Controls at ≥1 Month Post-Intervention

Kofotolis et al. (2008) [28] additionally looked at trunk muscle endurance 1 and 2 months post-intervention. At 1 month post-intervention, rhythmic stabilization exercises were significantly more effective than TENS at improving static flexion endurance (70 (11.6) vs. 58.2 (10.5), *p* < 0.05), static extension endurance (139.8 (4) vs. 83.1 (4.3), *p* < 0.05), dynamic flexion endurance (11.6 (2.1) vs. 7.8 (1.7), *p* < 0.05), and dynamic extension endurance (11.2 (1.3) vs. 9.2 (1.2), *p* < 0.05). At 2 months post-intervention, rhythmic stabilization exercises were significantly more effective than TENS at improving static flexion endurance (69.1 (14.9), *p* < 0.05), static extension endurance (140.3 (31.3) vs. 86.6 (32.1), *p* < 0.05), and dynamic extension endurance (11.3 (1.2) vs. 9.1 (1.3), *p* < 0.05).

#### 3.6.3. TENS versus Passive Controls at Post-Intervention

Kofotolis et al. (2008) [28] compared the effect of TENS with sham TENS for static and dynamic trunk muscle endurance in flexion and extension. TENS was significantly more effective than sham TENS at improving static flexion endurance (59.2 (5.5) vs. 53.4 (1.8), *p* < 0.05) but none of the other trunk endurance outcomes. 

#### 3.6.4. TENS versus Passive Controls at ≥1 Month Post-Intervention

Kofotolis et al. (2008) [28] additionally looked at trunk muscle endurance 1 and 2 months post-intervention. There were no significant differences between TENS and sham TENS for any of the trunk endurance outcomes at either time point.

#### 3.6.5. Mixed TENS versus Active Controls at Post-Intervention

**Figure 2:**
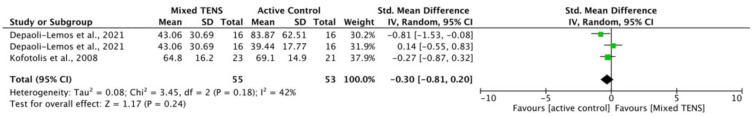
Static Trunk Flexion Endurance (post-intervention) [28,33].

**Figure 3:**
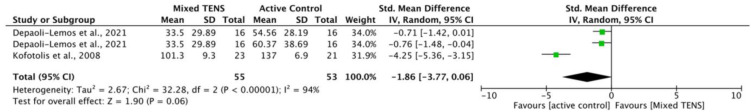
Static trunk extension endurance (post-intervention) [28,33].

Three comparisons across two studies [28,33] compared mixed TENS intervention with an active control for static trunk muscle endurance. For trunk *flexion* endurance, the meta-analysis revealed that mixed TENS was comparable to the active control (pooled SMD = −0.30, 95% CI = −0.81, 0.20). The comparisons made in this analysis had a moderate degree of heterogeneity (*I*^2^ = 42%). For trunk *extension* endurance, the meta-analysis revealed that mixed TENS was comparable to the active control (pooled SMD = −1.86, 95% CI = −3.77, 0.06). The comparisons made in this analysis had a high degree of heterogeneity (*I*^2^ = 94%). The results are presented below in Figure 2 and Figure 3. 

#### 3.6.6. Mixed TENS versus Active Controls at 1 Month Post-Intervention

**Figure 4:**
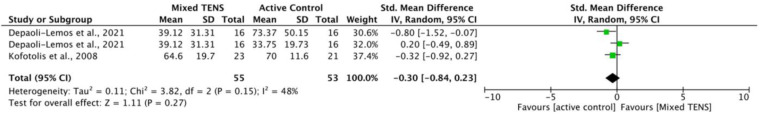
Static trunk flexion endurance (1 month post-intervention) [28,33].

**Figure 5:**
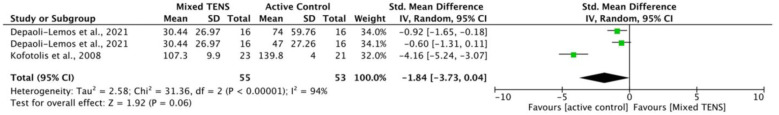
Static trunk extension endurance (1 month post-intervention) [28,33].

The same comparisons were made at 1 month post-intervention. For trunk *flexion* endurance, the meta-analysis revealed that mixed TENS was comparable to the active control (pooled SMD = −0.30, 95% CI = −0.84, 0.23). The comparisons made in this analysis had a moderate degree of heterogeneity (*I*^2^ = 48%). For trunk *extension* endurance, the meta-analysis revealed that mixed TENS was comparable to the active control (pooled SMD = −1.84, 95% CI = −3.73, 0.04). The comparisons made in this analysis had a high degree of heterogeneity (*I*^2^ = 94%). The results are presented below in Figure 4 and Figure 5. 

#### 3.6.7. Mixed TENS versus Passive Controls at Post-Intervention

Kofotolist et al. (2008) [28] compared the effect of TENS + rhythmic stabilization exercises with sham TENS for static and dynamic trunk endurance. At post-intervention, the mixed TENS group had greater static flexion endurance (71.4 (4.2) vs. 53.4 (1.8), *p* < 0.05), static extension endurance (137.0 (6.9) vs. 79.0 (6.8), *p* < 0.05), dynamic flexion endurance (12.1 (1.6) vs. 7.9 (1.2), *p* < 0.05), and dynamic extension endurance (11.4 (1.5) vs. 8.2 (0.9), *p* < 0.05) than the sham TENS group.

#### 3.6.8. Mixed TENS versus Passive Controls at ≥1 Month Post-Intervention

In the same study [28] at 1 month post-intervention, the mixed TENS group reported greater static flexion endurance (70.0 (11.6) vs. 51.7 (11.5), *p* < 0.05), static extension endurance (139.8 (4.0) vs. 78.6 (6.2), *p* < 0.05), dynamic flexion endurance (11.6 (2.1) vs. 7.6 (1.2), *p* < 0.05), and dynamic extension endurance (11.2 (1.3) vs. 8.8 (1.2), *p* < 0.05) than the sham TENS group. At 2 months post-intervention, the mixed TENS group continued to report greater static flexion endurance (69.1 (6.7) vs. 52.1 (8.5), *p* < 0.05), static extension endurance (140.3 (31.3) vs. 78.3 (29.9), *p* < 0.05), dynamic flexion endurance (11.7 (5.1) vs. 7.1 (3.0), *p* < 0.05), and dynamic extension endurance (11.3 (1.2) vs. 8.8 (1.2), *p* < 0.05) than the sham TENS group.

#### 3.6.9. EMS versus Active Controls at Post-Intervention

Dimer daLuz et al. (2019) [27] assessed the effect of NMES on static trunk muscle endurance. At post-intervention, there were no significant differences in either *flexion* or *extension* trunk muscle endurance between the NMES and core exercise groups.

#### 3.6.10. EMS versus Active Controls at ≥1 Month Post-Intervention

At 6 months post-intervention, Dimer daLuz et al. (2019) [27] reported no significant differences between the NMES and core-exercise groups for either *flexion* or *extension* trunk muscle endurance.

#### 3.6.11. EMS versus Passive Controls at Post-Intervention

Two studies [30,32] assessed the effect of their interventions on static trunk extensor muscle endurance. At post-intervention, Batistella et al. (2019) [32] reported significantly greater endurance in the Russian Current group compared to passive control (41.95 (12.09) vs. 31.21 (15.4), *p* = 0.0394), while Pelegrini et al. (2019) [30] found that the Aussie Current group had significantly greater endurance than the control group (48.38 (20.63) vs. 31.89 (8.81), *p* = 0.0191). 

#### 3.6.12. EMS versus Passive Controls at 1 Month Post-Intervention

At 1 month post-intervention, Pelegrini et al. (2019) [30] reported that the Aussie Current group had significantly greater endurance than the control group (40.12 (14.68) vs. 28.19 (6.72), *p* = 0.0176). Trunk muscle endurance was not reported on by Batistella et al. (2019) for this time point.

#### 3.6.13. Mixed EMS versus Active Controls at Post-Intervention

Dimer daLuz et al. (2019) [26] examined the effect of their interventions on static extensor endurance (Sorenson test) and reported significantly greater endurance in the mixed EMS group compared to the core exercise group (91.60 (23.77) vs. 60.70 (15.74), *p* < 0.05). They also assessed static trunk endurance, reporting significantly greater endurance in the mixed EMS group compared to the core exercise group (133.40 (53.02) vs. 46.30 (19.67), *p* < 0.05).

#### 3.6.14. Mixed EMS versus Active Controls at ≥1 Month Post-Intervention

Dimer daLuz et al. (2019) [27] re-examined trunk extensor endurance at 6 months post-intervention. There were no significant differences between the mixed EMS and core exercise groups. However, the mixed EMS group continued to demonstrate grater static trunk endurance than the core exercise group (83.60 (41.92) vs. 37.10 (13.70), *p* < 0.05).

### 3.7. Outcome: Paraspinal Muscle Thickness

#### 3.7.1. EMS versus Passive Controls at Post-Intervention

Two studies [30,32] assessed the effect of EMS on resting multifidus thickness. Batistella et al. (2019) [32] did not find significant between-group differences, while Pelegrini et al. (2019) [30] reported that the Aussie Current group had significantly greater thickness than the control group (4.51 (0.63) vs. 3.79 (0.58), *p* = 0.0049).

#### 3.7.2. EMS versus Passive Controls at 1 Month Post-Intervention

At 1 month post-intervention, Pelegrini et al. (2019) [30] reported that the Aussie Current group had significantly greater resting multifidus thickness than the control group (4.23 (0.60) vs. 3.71 (0.50), *p* = 0.0161). Multifidus thickness was not reported on by Batistella et al. (2019) [32] for this time point.

## 4. Discussion

This is the first systematic review to evaluate the effect of transcutaneous electrotherapies on multiple aspects of paraspinal muscle function in CLBP patients. Previously, Linzmeyer et al. (2022) [20] assessed the effect of EMS on paraspinal muscle strength and size in this population and concluded that NMES improved paraspinal muscle strength, with mixed effects on muscle size. Our review included the four studies that appeared in Linzmeyer et al. (2022) [20] plus an additional two studies that used WB-EMS, a mixed intervention. We also included three sensory electrotherapy studies for which spinal/trunk ROM was an outcome of interest. Furthermore, we separated stand-alone electrotherapy interventions from mixed interventions and made separate comparisons for active and passive controls. These distinctions are important, as they help researchers assess the specific effect of an intervention and help guide clinical practice.

Three studies looked at the effect of sensory electrotherapy on spinal ROM. At post-intervention, two of the studies found that participants who received sensory electrotherapy had better improvements to ROM than those who did not, while one study found that TENS—whether stand-alone or in conjunction with exercise—was less effective than exercise alone in improving ROM. This latter study (Kofotolis et al., 2008) [28] also reported that neither TENS nor mixed TENS were more effective than placebo TENS at improving ROM. In contrast to the results reported by Elserty et al. (2016) [33] and Lara-Palomo et al. (2013) [29], where sensory electrotherapy had a beneficial effect, the application of TENS by Kofotolis et al. (2008) [28] appears to have decreased treatment efficacy. This difference in outcome may in part be explained by differences in sensory application parameters between the studies. Elserty et al. (2016) [34] used conventional TENS (120 Hz) and Lara-Palomo et al. (2013) [29] used IFC, both of which are thought to provide immediate pain relief. In contrast, Kofotolis et al. (2008) [28] used low-frequency TENS (4 Hz), which has an onset of pain relief of at least 20 min post-treatment [15]. Given that participants in Kofotolis et al.’s mixed TENS group performed exercise 5 min after receiving TENS, it is possible that at the time of exercise, these participants had some lingering discomfort from the TENS treatment and were not yet able to benefit from it. The only study that re-examined outcomes at 1 and 2 months post-intervention—Kofotolis et al. [28]—found mixed TENS to be better than sham TENS at improving trunk extension ROM, although exercise alone was superior to all the other interventions at improving trunk ROM in general. 

EMS paired with strength training—whether superimposed or sequential—appears to have a beneficial effect on isometric trunk flexion and extension strength. The three studies that used this intervention and measured paraspinal muscle strength reported significant improvements in strength from baseline [17,26,31]. However, while WB-EMS was significantly more effective than no training [17], neither WB-EMS nor NMES *plus* stabilization exercises were more effective than conventional training and stabilization exercises alone, respectively, for improving trunk muscle strength [26,31]. These results are broadly in line with research that suggests that NMES improves maximal voluntary contractions (MVC) in healthy subjects [35]. 

Two studies [28,33] examined the effect of TENS on trunk muscle endurance. Based on the results, exercise is superior to TENS at improving endurance in flexion and extension, with benefits lasting at least 2 months post-intervention. The relative benefit of TENS compared to placebo TENS for improving trunk endurance appears negligible. With respect to the three mixed TENS interventions [28,33]—which were all LF TENS (≤10 Hz) [36] either proceeding or following exercise—the meta-analyses revealed no significant difference from active controls. There is no obvious mechanism by which TENS directly improves muscle function; although a by-product of LF TENS is the generation of a slight muscle twitch, this treatment aims to stimulate small-diameter afferent nerve fibers [15] and does not induce tetany [15]. However, mixed TENS was superior to placebo TENS for improving trunk endurance, likely due to the exercise component. 

EMS appears to generate short-term improvements in trunk endurance, and two of the three studies that tested EMS as a stand-alone intervention reported significant improvements in trunk endurance outcomes at post-intervention [30,32]. As with the strength outcomes, EMS was superior to passive but not active controls. When combined with exercise, EMS was superior to exercise alone at post-intervention. Importantly, in the study by Dimer da Luz et al. (2019) [27], both the EMS and mixed-EMS groups maintained improvements from baseline at long-term (6 months) follow-up, suggesting that EMS may have long-term effects on muscle physiology and/or neural activation in line with resistance training. Similar results were reported in a study of young men who underwent 24 weeks of resistance training followed by 24 weeks of de-training; after the de-training period, the participants reported significantly more strength than at baseline (*p* < 0.05) [37]. In contrast, the EMS groups in Batistella et al. (2020) [32] and Pelegrini et al. (2019) [30] reported a decrease in trunk endurance from post-intervention to 1 month post-intervention. There is no clear explanation for these differences, as all three studies were similar in terms of PICO variables: all contained a sample exclusively of women and all used medium-frequency electrotherapy for the same total number of sessions (12). 

Finally, though both Batistella et al. (2020) [32] and Pelegrini et al. (2019) [30] reported improvements in multifidus thickness at post-intervention following EMS, the *inter*- and *intra*group comparisons were statistically significant for Pelegrini et al. (2019) [30] alone. Additionally, the EMS groups in both studies had a decrease in multifidus thickness from post-intervention to 1 month post-intervention, suggesting a de-training effect that impacted the multifidus directly. A similar result was reported in a study of older adults who performed either trunk strengthening exercises or walking-balance exercises. Although the trunk strengthening group had significant increases in multifidus thickness at L4–L5 and L5-S1 after 12 weeks of training, these gains were lost after 6 weeks of de-training [38].

### Limitations

This systematic review had a number of limitations. First, while we were able to include ten studies in the qualitative synthesis, only two were eligible for meta-analysis due to the heterogeneity of the interventions and outcomes. As a result, we were only able to run a meta-analysis for two outcomes, limiting the scope of our findings. Additionally, there was considerable variety in the studies’ CLBP definition and inclusion criteria. Seven studies defined CLBP as low back pain of at least 3 months duration [26,27,29,30,32,33,34], two defined it as low back pain experienced on ≥50% of days in the previous 3 months [17,31], while one defined it as ‘chronic’—without specifying a baseline for symptom duration—whilst noting that participants were recruited from a pool of individuals who had LBP for at least 6 months [28]. In terms of inclusion criteria, three studies required participants to have at least a moderate amount of pain or disability at baseline [26,27,29] and the remaining seven did not [17,28,30,31,32,33,34]. Requiring study participants to have at least a moderate degree of pain or disability allows for minimum important changes (MIC) to be detected. This value has been reported on for a number of common self-reported LBP questionnaires: the Visual Analogue Scale (MIC = 15), Numerical Pain Rating Scale (MIC = 2), the Oswestry Disability Index (MIC = 20%), and the Roland Morris Disability Questionnaire (MIC = 5) [39]. Although self-reported questionnaires were not the focus of this review, participants in three studies [28,30,32] had baseline ODI scores of <20%, which may have limited the potential for their muscle strength and endurance to improve.

One strength of the studies included in this review is that they provided electrotherapy protocols in sufficient detail to allow for replication. However, four of the seven [17,26,27,28,31,33,34] studies that included an exercise component were vague in at least one aspect of the exercise protocol. Two studies did not clearly specify training volume [17,31], one did not clearly specify the parameters for exercise progression [28], and one neglected to provide the exercise program altogether [34]. Researchers should clearly define all aspects of an intervention protocol to allow for reproducibility.

Another limitation of this review is that there were relatively few studies included per outcome. Although many RCTs investigating the effect of transcutaneous electrotherapy on CLBP have been published, most have focused on self-reported outcomes such as pain and disability. Unfortunately, we were forced to exclude some studies investigating trunk-muscle-related outcomes for not meeting the inclusion criteria, including a lack of control group [40], insufficient treatment duration [41], and participant age [42]. If research is to paint a comprehensive picture of the effect of transcutaneous electrotherapy on trunk-muscle-related outcomes in CLBP patients, care should be taken to account for variables that can impede or confound the effects of an intervention. For example, 5–6 weeks is considered the minimum amount of time for NMES interventions to produce muscle hypertrophy [35,43], as strength gains made from shorter interventions will be the result of neural adaptations [43]. NMES researchers interested in paraspinal morphology outcomes can produce superior research by avoiding interventions of a shorter duration. With respect to age, smaller multifidus and erector spinae CSAs and higher levels of fat infiltration have been reported in older compared to younger individuals [44,45], with 60 years being identified as the age after which notable morphological changes occur [43]. Half of the studies in this review included participants > 60 years old [17,29,30,31,33], which may have blunted the intervention effect. Future studies may want to only include participants aged 18–60 years to limit the confounding effect of age. 

Finally, all the studies included in this systemic review had at least a moderate risk of bias, though this was due in large part to bias in the selection of the reported result, since none of the included studies published a protocol. Future RCTs can minimize the risk of bias by including the study protocol in their published report. 

## 5. Conclusions

In conclusion, there is limited evidence that EMS is superior to passive, but not active, controls for improving paraspinal muscle endurance in CLBP patients at post-intervention. There is limited evidence that EMS is comparable to active controls for improving paraspinal muscle endurance at ≥1 month post-intervention and conflicting evidence for its benefit compared to passive controls. There is also limited evidence that EMS *plus* exercise is superior to exercise alone for improving paraspinal muscle endurance in CLBP patients at post-intervention but not at ≥1 month post-intervention. There is limited evidence that neither TENS nor mixed TENS are superior to controls (active and passive) for improving paraspinal muscle endurance in CLBP patients at post-intervention and ≥1 month post-intervention. There is limited evidence that mixed NMES is superior to passive, but not active, controls for improving trunk muscle strength at post-intervention. There is conflicting evidence regarding the overall effect of EMS on paraspinal muscle thickness in CLBP patients. Finally, there is conflicting evidence about the overall effect of sensory electrotherapy on trunk/spinal ROM in CLBP patients, although higher pulse frequencies (80–120 Hz) [29,34] appear to be more effective than lower pulse frequencies (4 Hz) [28]. 

Our findings suggest that EMS is a viable alternative for improving paraspinal muscle endurance and may be particularly beneficial for patients with fear of movement or for whom exercise is not feasible. Although clinicians should prioritize the use of volitional exercise, when possible, EMS can form part of a multidisciplinary treatment strategy for CLBP alongside therapies such as exercise, mindfulness-based stress reduction [46], cognitive behavioral therapy [46], and oral supplementation with collagen peptides [47]. On the other hand, the results of this systematic review suggest there is no clinical basis for using TENS to improve paraspinal muscle endurance. To obtain a comprehensive understanding of the potential impact of transcutaneous electrotherapy for CLBP, future research can investigate outcomes that were not included in this review (ex: stand-alone EMS for paraspinal muscle strength, for which no eligible studies were found) or expand on the conflicting research into paraspinal muscle size and lumbar ROM. Where possible, researchers might try to adhere to the PICO variables of the studies included in this review in an intervention-dependent fashion. For example, the publication of more studies that used medium-frequency EMS and examined multifidus thickness would directly add to the existing body of research in this area and allow for future systematic reviews on this topic to be more robust.

## Data Availability

All extracted data are available upon request.

## Supplementary Materials

The following supporting information can be downloaded at https://www.mdpi.com/article/10.3390/jcm12144680/s1; Table S1: Risk of bias; Table S2: Characteristics of included studies; Table S3: Outcome: trunk/spinal ROM; Table S4: Outcome: paraspinal muscle strength/endurance; Table S5: Outcome: paraspinal muscle morphology.

## Appendix A. Sample Search Strategy (PubMed)

#1 Search: “Low Back Pain”[Mesh] OR “Back Pain”[Mesh:NoExp] OR “Hernia”[Mesh:NoExp] OR “Intervertebral Disc Displacement”[Mesh] OR “Intervertebral Disc Degeneration”[Mesh] OR “lumbosacral region” [Mesh] OR “Low back pain” OR “lumbago” OR “disc herniation” OR “intervertebral disc herniation” OR “disc degeneration” OR “degenerative disc” OR “lower back pain” OR “back ache” OR “backache” OR “backaches” OR “sacral pain” OR “lumbar pain” OR “lumbosacral pain” OR “LBP”
#2 Search: ‘Transcutaneous Electric Nerve Stimulation’[Mesh] OR ‘Electric Stimulation Therapy’[Mesh:NoExp] OR ‘Electric Stimulation’[Mesh:NoExp] OR “electric stimulation” OR “electrical stimulation” OR “electrostimulation” OR “TENS” OR “nerve stimulation” OR “electrotherapy” OR “interferential current” OR “electroanalgesia” OR “IFC” OR “neuromuscular electrical stimulation therapy” OR “NMES” OR “electromyostimulation” OR “EMS” OR “ES” OR “functional electrical stimulation” OR “FES” OR “Whole body electromyostimulation” OR “whole-body electromyostimulation” OR “WB-EMS”
#3 Search: (Animal[Mesh] NOT Human[Mesh])
#4 Search: #1 AND #2
#5: #4 NOT #3

## References

[1] Hoy D., Bain C., Williams G., March L., Brooks P., Blyth F., Woolf A., Vos T., Buchbinder R. (2012). A systematic review of the global prevalence of low back pain. Arthritis Rheum..

[2] Wong J.J., Côté P., Tricco A.C., Rosella L.C. (2019). Examining the effects of low back pain and mental health symptoms on healthcare utilisation and costs: A protocol for a population-based cohort study. BMJ Open.

[3] Martin B.I., Deyo R.A., Mirza S.K., Turner J.A., Comstock B.A., Hollingworth W., Sullivan S.D. (2008). Expenditures and Health Status Among Adults with Back and Neck Problems. JAMA.

[4] Hides J.A., Stanton W.R., Mcmahon S., Sims K., Richardson C.A. (2008). Effect of stabilization training on multifidus muscle cross-sectional area among young elite cricketers with low back pain. J. Orthop. Sports Phys. Ther..

[5] Danneels L.A., Coorevits P.L., Cools A.M., Vanderstraeten G.G., Cambier D.C., Witvrouw E.E., De Cuyper H.J. (2002). Differences in electromyographic activity in the multifidus muscle and the iliocostalis lumborum between healthy subjects and patients with sub-acute and chronic low back pain. Eur. Spine J..

[6] Stokes M., Hides J., Elliott J., Kiesel K., Hodges P. (2007). Rehabilitative ultrasound imaging of the posterior paraspinal muscles. J. Orthop. Sports Phys. Ther..

[7] Ledoux E., Dubois J.D., Descarreaux M. (2012). Physical and psychosocial predictors of functional trunk capacity in older adults with and without low back pain. J. Manip. Physiol. Ther..

[8] Dworkin R.H., Turk D.C., McDermott M.P., Pierce-Sandner S., Burke L.B., Cowan P., Farrar J.T., Hertz S., Raja S.N., Rappaport B.A. (2009). Interpreting the clinical importance of group differences in chronic pain clinical trials: IMMPACT recommendations. Pain.

[9] Deyo R.A., Battie M., Beurskens A.J., Bombardier C., Croft P., Koes B., Malmivaara A., Roland M., Von Korff M., Waddell G. (1998). Outcome measures for low back pain research: A proposal for standardized use. Spine.

[10] Vance C.G., Dailey D.L., Rakel B.A., Sluka K.A. (2014). Using TENS for pain control: The state of the evidence. Pain Manag..

[11] Airaksinen O., Brox J.I., Cedraschi C., Hildebrandt J., Klaber-Moffett J., Kovacs F., Mannion A.F., Reis S., Staal J.B., Ursin H. (2006). Chapter 4. European guidelines for the management of chronic nonspecific low back pain. Eur. Spine J..

[12] Kreiner D.S., Matz P., Bono C.M., Cho C.H., Easa J.E., Chiselli G., Ghogawala Z., Reitman C.A., Resnick D.K., Watters W.C. (2020). Guideline summary review: An evidence-based clinical guideline for the diagnosis and treatment of low back pain. Spine J..

[13] Jauregui J.J., Cherian J.J., Gwam C.U., Chughtai M., Mistry J.B., Elmallah R.K., Harwin S.F., Bhave A., Mont M.A. (2016). A meta-analysis of transcutaneous electrical nerve stimulation for chronic low back pain. Surg. Technol. Int..

[14] Wu L.C., Weng P.W., Chen C.H., Huang Y.Y., Tsuang Y.H., Chiang C.J. (2018). Literature review and meta-analysis of transcutaneous electrical nerve stimulation in treating chronic back pain. Reg. Anesth. Pain Med..

[15] Knight K.L., Draper D.O. (2013). Application procedures: Electrotherapy. Therapeutic Modalities: The Art and Science.

[16] Doucet B.M., Lam A., Griffin L. (2012). Neuromuscular electrical stimulation for skeletal muscle function. Yale J. Biol. Med..

[17] Weissenfels A., Teschler M., Willert S., Hettchen M., Fröhlich M., Kleinöder H., Kohl M., von Stengel S., Kemmler W. (2018). Effects of whole-body electromyostimulation on chronic nonspecific low back pain in adults: A randomized controlled study. J. Pain Res..

[18] Albright J., Allman R., Bonfiglio R.P., Conill A., Dobkin B., Guccione A.A., Hasson S.M., Russo R., Shekelle P., Susman J.L. (2001). Philadelphia panel evidence-based clinical practice guidelines on selected rehabilitation interventions for low back pain. Phys. Ther..

[19] Poitras S., Brosseau L. (2008). Evidence-informed management of chronic low back pain with transcutaneous electrical nerve stimulation, interferential current, electrical muscle stimulation, ultrasound, and thermotherapy. Spine J..

[20] Linzmeyer A., Coracini C.A., Bertolini G.R.F., Carvalho A.R. (2022). Effect of neuromuscular electrical stimulation on muscle function in chronic low back pain patients: Systematic review. BrJP.

[21] Page M.J., Moher D., Bossuyt P.M., Boutron I., Hoffmann T.C., Mulrow C.D., Shamseer L., Tetzlaff J.M., Akl E.A., Brennan S.E. (2021). PRISMA 2020 explanation and elaboration: Updated guidance and exemplars for reporting systematic reviews. BMJ.

[22] Furlan A.D., Malmivaara A., Chou R., Maher C.G., Deyo R.A., Schoene M., Bronfort G., van Tulder M.W., Editorial Board of the Cochrane Back, Neck Group (2015). 2015 updated method guideline for systematic reviews in the cochrane back and neck group. Spine.

[23] Simon J., McAuliffe M., Shamin F., Vuong N., Tahaei A. (2014). Discogenic low back pain. Phys. Med. Rehabil. Clin. N. Am..

[24] Macedo L.G., Maher C.G., Latimer J., Mcauley J.H. (2009). Motor control exercise for persistent, nonspecific low back pain: A systematic review. Phys. Ther..

[25] Sterne J.A.C., Savović J., Page M.J., Elbers R.G., Blencowe N.S., Boutron I., Cates C.J., Cheng H.-Y., Corbett M.S., Eldridge S.M. (2019). RoB 2: A revised tool for assessing risk of bias in randomised trials. BMJ.

[26] Alrwaily M., Schneider M., Sowa G., Timko M., Whitney S.L., Delitto A. (2019). Stabilization exercises combined with neuromuscular electrical stimulation for patients with chronic low back pain: A randomized controlled trial. Braz. J. Phys. Ther..

[27] Dimer da Luz R., da Silva Santos M., Steffen Evaldt A., da Silva Matos L., Boff Daitx R., Döhnert M.B. (2019). Neuromuscular electrical stimulation associated with core stability exercises in nonspecific postural low back pain: A randomized clinical trial. Muscles Ligaments Tendons J..

[28] Kofotolis N.D., Vlachopoulos S.P., Kellis E. (2008). Sequentially allocated clinical trial of rhythmic stabilization exercises and TENS in women with chronic low back pain. Clin. Rehabil..

[29] Lara-Palomo I.C., Aguilar-Ferrándiz M.E., Matarán-Peñarrocha G.A., Saavedra-Hernández M., Granero-Molina J., Fernández-Sola C., Castro-Sánchez A.M. (2013). Short-term effects of interferential current electro-massage in adults with chronic non-specific low back pain: A randomized controlled trial. Clin. Rehabil..

[30] Pelegrini A.C.A., Gasoto E., Bussolaro J.M., Segatti G., de Albuquerque C.E., Bertolini G.R.F. (2019). The analgesic action of Aussie current in women with non-specific chronic lumbar pain. Int. J. Ther. Rehabil..

[31] Weissenfels A., Wirtz N., Dörmann U., Kleinöder H., Donath H., Kohl M., Fröhlich M., von Stengel S., Kemmler W. (2019). Comparison of whole-body electromyostimulation versus recognized back-strengthening exercise training on chronic nonspecific low back pain: A randomized controlled study. Biomed Res. Int..

[32] Batistella C.E., Bidin F., Giacomelli I., Nunez M.A., Gasoto E., de Albuquerque C.E., Flores L.J.F., Bertolini G.R.F. (2020). Effects of the russian current in the treatment of low back pain in women: A randomized clinical trial. J. Bodyw. Mov. Ther..

[33] Depaoli Lemos V.J., Selau R.C., Blos C., Baptista Dohnert M., Boff Daitx R., de Almeida Brito V. (2021). Electroacupuncture and transcutaneous electrical nerve stimulation in chronic nonspecific low back pain: A blind randomized clinical trial. Muscles Ligaments Tendons J..

[34] Elserty N., Kattabei O., Elhafez H. (2016). Effect of fixed versus adjusted transcutaneous electrical nerve stimulation amplitude on chronic mechanical low back pain. J. Altern. Complement. Med..

[35] Herzig D., Maffiuletti N.A., Eser P. (2015). The application of neuromuscular electrical stimulation training in various non-neurologic patient populations: A narrative review. PM&R.

[36] Montenegro E.J., Guimarães de Alencar G., Rocha de Siqueira G., Guerino M.R., Maia J.N., Araújo de Oliveira D. (2016). Effect of low frequency transcutaneous electrical nerve stimulation of TE5 (waiguan) and PC6 (neiguan) acupoints on cold-induced pain. J. Phys. Ther. Sci..

[37] Lo M.S., Lin L.L.C., Yao W.J., Ma M.C. (2011). Training and detraining effects of the resistance vs. endurance program on body composition, body size, and physical performance in young men. J. Strength Cond. Res..

[38] Shahtahmassebi B., Hebert J.J., Hecimovich M., Fairchild T.J. (2019). Trunk exercise training improves muscle size, strength, and function in older adults: A randomized controlled trial. Scand. J. Med. Sci. Sports.

[39] Ostelo R.W.J.G., Deyo R.A., Stratford P., Waddell G., Croft P., Von Korff M., Bouter L.M., de Vet H.C. (2008). Interpreting change scores for pain and functional status in low back pain: Towards international consensus regarding minimal important change. Spine.

[40] Coghlan S., Crowe L., McCarthypersson U., Minogue C., Caulfield B. Neuromuscular electrical stimulation training results in enhanced activation of spinal stabilizing muscles during spinal loading and improvements in pain ratings. Proceedings of the Annual International Conference of the IEEE Engineering in Medicine and Biology Society.

[41] Guo P., Wang J.-W., Tong A. (2018). Therapeutic effectiveness of neuromuscular electrical stimulation for treating patients with chronic low back pain. Medicine.

[42] Hicks G.E., Sions J.M., Velasco T.O., Manal T.J. (2016). Trunk muscle training augmented with neuromuscular electrical stimulation appears to improve function in older adults with chronic low back pain: A randomized preliminary trial. Clin. J. Pain.

[43] Vanderthommen M., Duchateau J. (2007). Electrical stimulation as a modality to improve performance of the neuromuscular system. Exerc. Sport Sci. Rev..

[44] Shahidi B., Parra C.L., Berry D.B., Hubbard J.C., Gombatto S., Zlomislic V., Todd Allen R., Hughes-Austin J., Garfin S., Ward S.R. (2017). Contribution of lumbar spine pathology and age to paraspinal muscle size and fatty infiltration. Spine.

[45] Huang R., Pan F., Kong C., Lu S. (2022). Age- and sex-dependent differences in the morphology and composition of paraspinal muscles between subjects with and without lumbar degenerative diseases. BMC Musculoskelet. Disord..

[46] Qaseem A., Wilt T.J., McLean R.M., Forciea M.A., Denberg T.D., Barry M.J., Boyd C., Chow R.D., Fitterman N., Clinical Guidelines Committee of the American College of Physicians (2017). Noninvasive treatments for acute, subacute, and chronic low back pain: A clinical practice guideline from the American College of Physicians. Ann. Intern. Med..

[47] Farì G., Santagati D., Pignatelli G., Scacco V., Renna D., Cascarano G., Vendola F., Bianchi F.P., Fiore P., Ranieri M. (2022). Collagen peptides, in association with vitamin c, sodium hyaluronate, manganese and copper, as part of the rehabilitation project in the treatment of chronic low back pain. Endocr. Metab. Immune Disord. Drug Targets.

