# Supplementary material for: The Effect of Transcutaneous Electrotherapy on Lumbar Range of Motion and Paraspinal Muscle Characteristics in Chronic Low Back Pain Patients: A Systematic Review and Meta-Analysis
Source: J Clin Med. 2023 Jul 14;12(14):4680. doi: 10.3390/jcm12144680 (PMC10380811; doi:10.3390/jcm12144680)
Supplement: Supplementary file 1 [file jcm-12-04680-s001.zip › jcm-2478784-supplementary.pdf]

Supplementary Table S1 – Risk of Bias

Included Study	Outcome Variable	Comparison	Bias from the Randomization Process	Bias due to deviations from intended interventions	Bias due to missing outcome data	Bias in measurement of the outcome	Bias in selection of the reported result	Overall judgment
Alrwaily et al., 2019 [26]	Trunk muscle strength	NMES + exercise (mixed) vs. exercise (active control)	Low	Low	Low	Some concerns	Some concerns	Some concerns
Batistella et al., 2020 [32]	Trunk muscle endurance	Russian current vs. passive control	Low	Low	Low	High	High	High
	Paraspinal muscle thickness	Russian current vs. passive control	Low	Low	Low	High	High	High
Depaoli-Lemos et al., 2021 [33]	Trunk muscle endurance	TENS + exercise (mixed) vs. exercise (active control)	Low	Low	Low	High	High	High
Dimer daLuz et al., 2019 [27]	Trunk muscle endurance	NMES vs. exercise (active control)	Low	Low	Low	Low	Some concerns	Some concerns
	Trunk muscle endurance	NMES + exercise (mixed) vs. exercise (active control)	Low	Low	Low	Low	Some concerns	Some concerns
Elserty et al., 2016 [34]	Spinal ROM	TENS vs. exercise (active control)	Low	High	High	Low	Some concerns	High
Kofotolis et al., 2008 [28]	Lumbar ROM	TENS vs. exercise (active control)	Some concerns	Low	Low	Low	Some concerns	Some concerns
	Trunk muscle endurance	TENS vs. exercise (active control)	Some concerns	Low	Low	Low	Some concerns	Some concerns
	Lumbar ROM	TENS vs. placebo TENS (passive control)	Some concerns	Low	Low	Low	Some concerns	Some concerns
	Trunk muscle endurance	TENS vs. placebo TENS (passive control)	Some concerns	Low	Low	Low	Some concerns	Some concerns
	Lumbar ROM	TENS + exercise (mixed) vs. exercise (active control)	Some concerns	Low	Low	Low	Some concerns	Some concerns
	Trunk muscle endurance	TENS + exercise (mixed) vs. exercise (active control)	Some concerns	Low	Low	Low	Some concerns	Some concerns
	Lumbar ROM	TENS + exercise (mixed) vs. placebo TENS (passive control)	Some concerns	Low	Low	Low	Some concerns	Some concerns
	Trunk muscle endurance	TENS + exercise (mixed) vs. placebo TENS (passive control)	Some concerns	Low	Low	Low	Some concerns	Some concerns

LaraPalomo et al., 2013 [29]	Lumbar ROM	IFC vs. superficial massage (active control)	Low	Low	Low	Low	Some conecrns	Some concerns
Pelegrini et al., 2019 [30]	Trunk muscle endurance	Aussie Current vs. passive control	Some concerns	Low	Low	Low	Some concerns	Some concerns
	Paraspinal muscle thickness	Aussie Current vs. passive control	Some concerns	Low	Low	Low	Some concerns	Some concerns
Weissenfels et al., 2018 [17]	Trunk muscle strength	WB-EMS vs. Passive control	Low	Low	Low	Low	Some concerns	Some concerns
Weissenfels et al., 2019 [31]	Trunk muscle strength	WB-EMS vs. Exercise (active control)	Low	Low	Low	Low	Some concerns	Some concerns

Supplementary Table S2 – Characteristics of Included Studies

Study	Participants	Study Arms	Treatment	Duration	Outcomes	Time points
Alrwaily et al., 2019 [26]	Individuals aged 18-60 with CLBP, BMI < 34, NPRS ≥ 3, MODI ≥ 20% (n=26; 4 dropouts)<br><br>Age: STAB = 38.33 (11.3); SNMES = 33.4 (9)<br><br>Sex: STAB = 4 men, 11 women; SNMES = 7 men, 8 women<br><br>BMI: STAB = 25.89 (3.8); SNMES = 26.47 (2.9)	1. STAB (n=13)<br>2. SNMES (n=13)	1. 5-6 cycles of cat-camel, followed by supine & standing postures with abdominal bracing, bridging, supine leg-lifts, quadruped, and side-support exercises. Positions were held for 4-8s and progressed once target reps were reached.<br>2. NMES: EMPI 300 unit; 75pps; 250us; 4s ramp time, 6s stimulation period, 50s rest. Electrodes were applied to the lumbar paraspinal muscles bilaterally. Participants were told that the stronger the current, the better. They performed active trunk extension when they felt the current ramp up, and rested when the current ramped down. This was followed by the stabilization program described above.	1. 20 minutes/ session, 2x/week for 6 weeks<br>2. 40 minutes/ session, 2x/week for 6 weeks	Pain (triple NPRS); disability (MODI); fear-avoidance for physical activity and work (FABQ-PA & FABQ-W); paraspinal muscle strength (Biodex 3 Pro dynamometer); self-reported NMES tolerability (NMES group only)	Baseline; during final session (week 6); week 10 (NPRS & MODI only)
Batistella et al., 2020 [32]	Sedentary women with CNSLBP (n=23; 1 exclusion)<br><br>Age: 21.5 (2.3)<br><br>BMI: 22.3 (3.8)	1. RC (n=11)<br>2. C (n=12)	1. Russian current: two-channel; Ibramed unit; 2500Hz frequency with 50Hz modulation; 2s ramp, 8s on, 12s off time. Electrodes were placed 3cm lateral to the SPs of T12 & S1. Intensity was	1. 20 minutes/session, 3 sessions/ week for 4 weeks.<br>2. Two months	Pain (VAS); pain pressure threshold (Kratos algometer); disability (ODI); paraspinal muscle endurance; resting	Baseline; post-intervention; 1 month post-intervention

Study	Participants	Study Arms	Treatment	Duration	Outcomes	Time points
			increased so that a non-painful muscular contraction was perceived through palpation.<br>2. No intervention. Group was asked to not perform any physical or medicinal activities during the study period.		multifidus thickness (US)	
Depaoli-Lemos et al., 2021 [33]	Individuals 18-70y with CLBP (n=48)<br><br>Age: EXCITENS = 52.5 (12.42); EXCIEA = 45.37 (13.51); EXCI = 50.81 (12.96)<br><br>Sex: EXCITENS = 3 male, 13 female; EXCIEA = 6 male, 10 female; EXCI = 5 male, 11 female<br><br>BMI: EXCITENS = 28.95 (4.32); EXCIEA = 27.36 (4); EXCI = 27.29 (3.11)	1. EXCI (n=16)<br>2. EXCITENS (n=16)<br>3. EXCIEA (n=16)	1. Exercise: stretches for the posterior chain muscles, isometric strengthening exercises for the core (supine bridge, single leg supine bridge, side plank, prone plank).<br>2. Exercise: as above. Followed by electroacupuncture: Sikuro DS100jr unit, two channels with four stimulator cables; 25x40mm needles used at bladder meridian points B22 (L1) & B26 (L5). Signal 1, continuous pulse train, 10Hz. Intensity was increased to tolerance.<br>3. Exercise: as above. Followed by TENS: two-channel Ibramed unit, 250us; 10Hz. Four electrodes placed bilaterally on the paravertebral musculature; current intensity increased to participant's tolerance.	1. 50 minutes/session, 3 sessions/week for 4 weeks<br>2. 50 minutes/session, 3 sessions/week for 4 weeks (including 20 minutes TENS)<br>3. 50 minutes/session, 3 sessions/week for 4 weeks (including 20 minutes EA)	Pain (VAS); function (Roland-Morris); posterior chain flexibility (Wells Bench test); static trunk flexion endurance; static trunk extension endurance (Sorenson test); side bridge	Baseline, post-intervention, 1-month post-intervention
Dimer daLuz et al., 2019 [27]	Females 18-36y with CNSLBP, not engaging in regular physical activity, VAS > 4/10 (n=30; 2 withdrawals)<br><br>Age: CORE = 26.4 (3.41); NMES = 27.1	1. CORE (n=10)<br>2. NMES (n=10)<br>3. COMB (n=10)	1. CORE: 4 exercises per session, each maintained for 10s. 10 reps were performed with a 20s rest between sets and 1 minute rest between exercises. Exercises: prone bridge, supine, side bridge, bird-dog + progressions.	1. ~ 7 minutes of training time (not including rest periods)/session, 3x/ week for 4 weeks<br>2. 25 minutes/session 3x/ week for 4 weeks<br>3. 25 minutes/session 3x/ week for 4 weeks	Pain (VAS); disability (ODI); function (Roland-Morris); hamstring flexibility; static trunk endurance; back extensor endurance (Sorenson test); side bridge; prone instability	Baseline, post-intervention, 6-months post-intervention

Study	Participants	Study Arms	Treatment	Duration	Outcomes	Time points
	(4.95); COMB = 25.5 (5.28)<br><br>BMI: CORE = 22.56 (3.35); NMES = 27.74 (5.36); COMB = 25.79 (5.5)		2. NMES: Program targeted gluteus maximums and medius, rectus abdominus, transversus abdominus. Pre-calibrated mid-frequency 10-channel Neruodyn unit; 2500 Hz carrier frequency; 1s ramp time, 10s on, 20s off. Negative electrodes were positioned on the motor points, and positive electrodes were positioned proximal or distal to the muscle belly. Stimulus started at 5 Hz for 5 minutes, increased to 35 Hz for 10 minutes, and then 80 Hz for 10 minutes. Intensity was increased to the max needed to produce a strong, visible muscle contraction without causing discomfort to the participant.<br>3. COMB. ES was synchronized to CORE exercises.			
Elserty et al., 2016 [34]	CLBP patients 20-50y, without radiating pain (n=40; 75 withdrawals)<br><br>Age: EXCI = 34.93 (8.56); FTENS = 35.73 (8.01); ATENS = 35.13 (8.4)<br><br>Sex: 14 men (31%), 31 women (69%)	1. EXCI (n=15)<br>2. FTENS (n=15)<br>3. ATENS (n=15)	1. EXCI: progression of bridging and quadruped exercises.<br>2. FTENS: symmetric biphasic current, pulse duration 100ms, frequency 120Hz. Electrodes were placed bilaterally at the level of the lumbar vertebrae. Amplitude was increased until patients felt a comfortable tingling sensation + EXCI.<br>3. ATENS: symmetric biphasic current, pulse duration 100ms, frequency 120Hz. Electrodes were placed bilaterally at the level of the lumbar vertebrae. Amplitude was increased until patients felt a comfortable tingling sensation. At 5-minute intervals, participants were asked if the sensation had	1. Length of sessions not described, 3x/ week for 4 weeks<br>2. TENS for 40 min + EXCI, 3x/week for 4 weeks<br>3. TENS for 40 min + EXCI, 3x/week for 4 weeks	Pain (VAS); disability (ODI); spinal ROM in flexion/extension (dual inclinometer)	Baseline, post-intervention

Study	Participants	Study Arms	Treatment	Duration	Outcomes	Time points
			faded; if so, the amplitude was increased until the tingling reappeared + EXCI.			
Kofotolis et al., 2008 [28]	Women with CLBP, who had both unsuccessful resting periods for 6 months prior and unsuccessful previous therapy (n=88; 4 withdrawals)<br><br>Age: RS = 41 (5.5); TENS = 41.2 (5); COMB = 37.5 (8.6); PTENS = 42.2 (7.8)<br><br>BMI: RS = 24.9 (1.2); TENS = 24.6 (1); COMB = 24.3 (1.4); PTENS = 23.8 (1.7)	1. RS (n=23)<br>2. TENS (n=23)<br>3. **COMB** (n=21)<br>4. PTENS (n=21)	1. RS: Alternating isometric trunk flexion-extension exercises against resistance for 10 seconds. Participants performed 3x15 at max resistance provided by a physical therapist. 30s rest between reps (each pattern), 60s rest between sets. Intensity progression was made according to PNF principles based on participants' mobility progress.<br>2. TENS: 120 Z unit (ITO, Tokyo, Japan); pulse duration 200us, frequency 4Hz, intensity 'strong but comfortable'. Four rubber electrodes (2 x 3 cm) from a dual channel unit were applied to the thoracolumbar fascia and 10cm proximal, along the midline of the muscle.<br>3. **COMB**: TENS followed by RS.<br>4. PTENS: The same unit was used as for TENS, but the internal circuit was disconnected by the manufacturer.	1. 30-45 min, 5x/ week for 4 weeks<br>2. 40-45 min, 5x/week for 4 weeks<br>3. 20 min TENS, 5 min rest, 20 min RS (total 45 min), 5x/ week for 4 weeks<br>4. 40-45 min, 5x/week for 4 weeks	Pain (Borg Verbal Rating Pain Scale); disability (ODI); trunk ROM in flexion/extension (flexicurve technique); dynamic flexion endurance (curl-up); dynamic extension endurance (modified Sorenson back extension test); Static flexion endurance (curl-up); static extension endurance (modified Sorenson back extension test)	Baseline, post-intervention, 1-month post, 2-month post
LaraPalomo et al., 2013 [29]	Individuals 18-65y with CLBP, ≥ 4 on Roland-Morris, not undergoing another physical therapy intervention, with an inability to achieve lumbar muscle flexion-relaxation in trunk flexion (n=61; 1 lost to follow-up)	1. IFC (n=30)<br>2. SM (n=31)	1. 4000Hz carrier frequency, 80Hz amplitude modulation, constant voltage. Bipolar application with 2 electrodes to which sponges were fitted. The sponges moved over the lumbar and thoraco-lumbar regions. Intensity was increased to between 30-50mA, always below pain threshold.	1. 30 minutes/session, 2x/ week for 10 weeks<br>2. 20 minutes/session, 2x/ week for 10 weeks	Pain (VAS); disability (ODI); function (Roland-Morris); kinesiophobia (TSK); quality of life (SF-36); isometric abdominal resistance (McQuade test); side bridge test; trunk anteflexion ROM	Baseline, post-intervention.

Study	Participants	Study Arms	Treatment	Duration	Outcomes	Time points
	Age: IFC = 50 (15); SM = 47 (13)<br><br>Sex: IFC = 21 women, 9 men; SM = 20 women, 11 men.		2. Massage consisting of effleurage, petrissage, and skin rolling.			
Pelegrini et al., 2019 [30]	Patients with low back pain for at least 3 months, aged 19-70y, (n=24, no dropouts)<br><br>Age: 20.4 (1.8) y<br><br>Sex: 100% women	1. AC (n=12)<br>2. C (n=12)	1. Treatment with Neurodyn Ibramed device. Frequency 1 kHz, modulation 50 Hz, burst duration 4ms, ramp time 1s, maintained for 8s, rest 10s. Intensity increased until visible (but not painful) involuntary muscular contraction was achieved. If accommodation phenomenon was achieved, the therapist increased the amplitude of the current<br>2. Participants agreed to not participate in any therapeutic activity for 4 weeks.	1. 20 minutes, 3x / week for 4 weeks<br>2. No treatment for 4 weeks	Pain (VAS, McGill Pain Questionnaire); disability (ODI); trunk extensor endurance (horizontal board test aka Sorenson test); resting multifidus thickness (ultrasound)	Baseline, post-intervention, 1-month post-intervention
Weissenfels et al., 2018 [17]	Patients with nonspecific chronic low back pain for at least 3 months (aged 40-70y) (n=30)<br><br>Age: WB-EMS = 54.6 (5.7); C = 59.4 (7.7)<br><br>Total body fat %: WB-EMS = 25.4 (9.3) for men, 31.3 (8.4) for women; C: 29.9 (5.4) for men, 35.5 (9.1) for women	1. WB-EMS (n=15)<br>2. C (n=15)	1. Bipolar electric current with a frequency of 85 Hz, an impulse width of 350 µs, a rectangular mode and an interval of 6 seconds stimulation and 4 seconds of rest once a week for 20 minutes. During the stimulation phase, participants performed low-amplitude movements specifically dedicated to LBP. The participants completed one to three sets with six repetitions of six easy movements in a minor range of motion (eg, dynamic squatting with knee ankle ≥120°) to keep the effect of the voluntary exercise itself as low as possible. The intensity of the stimulation was regulated using the BORG CR	1. 12 min for 1st session, 14 for 2nd, 16 for 3rd, 18 for 4th, 20 min for last 8 weeks. 1 session/ week for a total of 12 weeks<br>2. No specific intervention for 12 weeks	Average pain Intensity (NPRS); isometric trunk flexion strength; isometric trunk extension strength	For pain: baseline (4 weeks prior to intervention); post-intervention (last 4 weeks of intervention)<br><br>For strength: baseline, post-intervention

Study	Participants	Study Arms	Treatment	Duration	Outcomes	Time points
			10 scale. Subjects were requested to exercise at a rate of perceived exertion (RPE) between "hard (5)" and "very hard (7)". In the first session current intensity was individually adapted in close interaction with the participants and saved to generate a fast and valid setting during the following sessions.<br>2. The CG was asked to maintain its usual lifestyle. Participants of this group were regularly contacted by phone and asked about their current status and lifestyle changes.			
Weissenfels et al., 2019 [31]	Patients with nonspecific chronic low back pain for at least 3 months (aged 40-70y) (n=110)<br><br>Age: WB-EMS = 54.4 (7.4); CT = 57.4 (7.6)<br><br>Sex: WB-EMS = 20m, 35w; CT = 17m, 38w<br><br>Total body fat %: WB-EMS = 25.1 (8.9) for men, 32.9 (8.7) for women; CT = 23.4 (4.3) for men, 35 (8.2) for women;	1. WB-EMS (n-55)<br>2. CT (n=55)	1. Bipolar electric current with a frequency of 85 Hz, an impulse width of 350 µs, a rectangular mode and an interval of 6 seconds stimulation and 4 seconds of rest once a week for 20 minutes. Participants performed the following exercises: squat with latissimus pulleys, butterfly reverse, straight pullovers with trunk flexion, standing trunk flexion; one-legged stand with biceps curl; side step with weight shift and biceps curl. With the exception of the first week, participants were instructed to perform exercises at an RPE between 'strong' and 'very strong'<br>2. Participants performed conventional back strengthening / core stabilization exercises described in various meta-analyses. After 15 minutes	1. 12 min for 1st session, 14 for 2nd, 16 for 3rd, 18 for 4th, 20 min for last 8 weeks. 1 session/ week for a total of 12 weeks<br>1. 45 minutes per week for 12 weeks	Average pain Intensity (NPRS); isometric trunk flexion strength (Back-Check 607); isometric trunk extension strength (Back-Check 607)	For pain: baseline (4 weeks prior to intervention); post-intervention (last 4 weeks of intervention)<br><br>For strength: baseline, post-intervention

Study	Participants	Study Arms	Treatment	Duration	Outcomes	Time points
			warm-up, 10 trunk strengthening exercises were performed in a circle for 30 minutes. The circle repeated twice, with 50s work and 25s break between exercises.			

<u>LEGEND</u>: CLBP = chronic low back pain; CNSLP = chronic non-specific low back pain; BMI = body mass index; CSA = cross-sectional area; ROM = range of motion; TENS = transcutaneous electrical nerve stimulation; NMES = neuromuscular electrical nerve stimulation; WB-EMS = whole-body electromyostimulation; IFC = interferential current therapy; NPRS = Numerical Pain Rating Scale; VAS = Visual Analog Scale; ODI = Oswestry Disability Index; MODI = Modified Oswestry Disability Index; FABQ-W = Fear-Avoidance Beliefs Questionnaire – Work; FABQ-PA = Fear-Avoidance Beliefs Questionnaire – Physical Activity; STAB = stabilization exercise; SNMES = stabilization exercises + NMES; RC = Russian current; C = control; EXCI = exercise; EXCITENS = exercise + TENS; EXCIEA = exercise + electroacupuncture; CORE = core exercise; COMB = core exercise + NMES; FTENS = fixed TENS + exercise; ATENS = adjusted TENS + exercise; RS = rhythmic stabilization; **COMB** = rhythmic stabilization + TENS: PTENS = placebo TENS; SM = superficial massage; AC = Aussie current; CT = conventional training

Supplementary Table S3 – Outcome: Trunk / Spinal ROM

Study	Groups	Outcome/Tool	Result: post-intervention	Result: 1-month post	Result: ≥2-month post
Elserty et al., 2016 [34]	1. EXCI (n=15)<br>2. FTENS (n=15)<br>3. ATENS (n=15)	Spinal ROM (dual inclinometer)	<u>Spinal Flexion ROM</u><br>EXCI group changed from 27.2 (8.78) to 43.12 (4.66)<br><br>FTENS group changed from 27.07 (6.47) to 50.53 (5.67).<br><br>ATENS group changed from 26.67 (6.03) to 51.0 (5.0).<br><br>There was a significant between-group difference at post- intervention (**p = 0.0001**). Fisher least-significant difference test revealed greater improvements in both TENS groups compared to the EXCI group.<br><br><u>Spinal Extension ROM</u><br>EXCI group changed from 11.13 (3.36) to 17.07 (2.49)<br><br>FTENS group changed from 10.87 (2.88) to 19.27 (3.28).	NA	NA

			ATENS group changed from 10.4 (2.82) to 19.73 (2.4).<br><br>There was a significant between-group difference at post- intervention (**p = 0.026**). Fisher least-significant difference test revealed greater improvements in both TENS groups compared to the EXCI group.		
Kofotolis et al., 2008 [28]	1. RS (n=23)<br>2. TENS (n=23)<br>3. COMB (n=21)<br>4. PTENS (n=21)	Trunk ROM (flexicurve technique)	<u>Trunk Flexion ROM</u><br>RS group improved from 60.3 (7.8) to 66.4 (5.4), **p < 0.05.**<br><br>TENS group changed from 60.5 (3.2) to 61.1 (3.9)<br><br>COMB group changed from 59.8 (2.3) to 61.3 (3.2)<br><br>PTENS group changed from 61.3 (1.4) to 60.4 (3.8)<br><br>There were significant differences (**p < 0.05**) between RS & PTENS, RS & TENS, RS & COMB (all in favor of RS.<br><br><u>Trunk Extension ROM</u><br>RS group improved from 23.4 (2.2) to 27.4 (1.1), **p < 0.05.**<br><br>TENS group changed from 23.7 (2.0) to 24.5 (2.7)<br><br>COMB group changed from 23.8 (2.0) to 25.4 (2.9)<br><br>PTENS group changed from 22.8 (1.0) to 22.9 (4.9)<br><br>There were significant differences (**p < 0.05**)	<u>Trunk Flexion ROM</u><br>RS: 73.8 (8.2). Significant improvement from baseline to 1-month post-intervention (**p < 0.05**).<br><br>TENS: 62.5 (15.3)<br><br>COMB: 61.7 (15.8)<br><br>PTENS: 61.6 (15.3)<br><br>There were significant differences (**p < 0.05**) between RS & PTENS, RS & TENS, RS & COMB (all in favor of RS.<br><br><u>Trunk Extension ROM</u><br>RS: 28.9 (1.7). Significant improvement from baseline to 1-month post-intervention (**p < 0.05**).<br><br>TENS: 24.5 (2.9)<br><br>COMB: 25.5 (4.0). Significant improvement from baseline to 1-month post-intervention (**p < 0.05**).<br><br>PTENS: 22.0 (4.6)<br><br>There were significant differences (**p < 0.05**)	<u>Trunk Flexion ROM</u><br>RS: 75.7 (10.2). Significant improvement from baseline to 1-month post-intervention (**p < 0.05**).<br><br>TENS: 62.7 (1.5)<br><br>COMB: 63.7 (3.5)<br><br>PTENS: 62.3 (6.4)<br><br>There were significant differences (**p < 0.05**) between RS & PTENS, RS & TENS, RS & COMB (all in favor of RS.<br><br><u>Trunk Extension ROM</u><br>RS: 29.2 (2.1). Significant improvement from baseline to 1-month post-intervention (**p < 0.05**).<br><br>TENS: 24.9 (3.0)<br><br>COMB: 26.0 (3.6). Significant improvement from baseline to 1-month post-intervention (**p < 0.05**).<br><br>PTENS: 23.0 (4.1)<br><br>There were significant differences (**p < 0.05**)

			between RS & PTENS, RS & TENS (both in favor of RS.	between RS & PTENS, RS & TENS, RS & COMB (all in favor of RS, and between COMB & PTENS (in favor of COMB).	between RS & PTENS, RS & TENS, RS & COMB (all in favor of RS, and between COMB & PTENS (in favor of COMB).
LaraPalomo et al., 2013 [29]	1. IFC (n=30)<br>2. SM (n=31)	Trunk Flexion ROM (tape measure)	IFC group improved from baseline (MD = 3.01, 95% CI = 1.84, 4.16, p = 0.004).<br><br>SM group improved from baseline (MD = 1.48, 95% CI = 0.35, 2.62, p = 0.048).<br><br>There was an insignificant between-group difference in score change (MD = -1.12, 95% CI = -3.79, -1.54, p = 0.062) in favour of IFC.	NA	NA

<u>LEGEND</u>: ROM = range of motion; TENS = transcutaneous electrical nerve stimulation; IFC = interferential current; EXCI = exercise; FTENS = fixed TENS + exercise; ATENS = adjusted TENS + exercise; RS = rhythmic stabilization; COMB = rhythmic stabilization + TENS: PTENS = placebo TENS; SM = superficial massage; MD = mean difference

Supplementary Table S4 – Outcome: Paraspinal Muscle Strength / Endurance

Study	Groups	Outcome/Tool	Result: post-intervention	Result: 1-month post	Result: ≥2-month post
Alrwaily et al., 2019 [26]	1. STAB (n=13)<br>2. SNMES (n=13)	Paraspinal muscle strength (Biodex 3 Pro dynamometer)	STAB group improved from 117.29 (57.7) Nm to 162.30 (55.2) Nm, Δ 45.01, **p < 0.05**.<br><br>SNMES group improved from 154.49 (59.1) Nm to 175.80 (58.4) Nm, Δ 21.31, **p < 0.05**.<br><br>Δ in strength was not significantly greater in the STAB group (MD = -13.51, 95% CI = -56.0, 28.98).	NA	NA
Batistella et al., 2020 [32]	1. RC (n=11)<br>2. C (n=12)	Paraspinal muscle endurance	RC group improved from 29.37 (10.22) to 41.95 (12.09), **p=0.0208**.<br><br>C group changed from 35.8 (18.49) to 31.21 (15.4), p > 0.05.	RC group: 31.58 (9.4).<br><br>C group: 28.58 (14.24).<br><br>There was no significant between-group difference at 1-month post-intervention (p=0.288).	NA

			There was a significant difference at post-intervention between the groups (**p=0.0394**).		
Depaoli-Lemos et al., 2021 [33]	1. EXCITENS (n=16)<br>2. EXCIEA (n=16)<br>3. EXCI (n=16)	Static trunk flexion endurance; static trunk extension endurance (Sorenson test)	<u>Static trunk flexion endurance:</u><br>EXCITENS group improved from 17.94 (12.21) to 43.06 (30.69), **p < 0.05**.<br><br>EXCIEA group improved from 28.81 (31.33) to 83.87 (62.51), **p < 0.05**.<br><br>EXCI group improved from 24.37 (18.52) to 39.44 (17.77), **p < 0.05**.<br><br>There were significant between-group differences (**p = 0.006**) between EXCITENS & EXCIEA, and between EXCIEA & EXCI (both in favour of EXCIEA).<br><br><u>Sorenson test:</u><br>EXCITENS group changed from 22.62 (15.89) to 33.5 (29.89), p > 0.05.<br><br>EXCIEA group improved from 30.5 (20.28) to 60.37 (38.69), **p < 0.05**.<br><br>EXCI group improved from 37.94 (25.24) to 54.56 (28.19), **p < 0.05**.<br><br>There were no significant between-group differences.	<u>Static trunk flexion endurance:</u><br>EXCITENS: 39.12 (31.31). Significant improvement from baseline to 1-month post (**p < 0.05**).<br><br>EXCIEA: 73.37 (50.15). Significant improvement from baseline to 1-month post (**p < 0.05**).<br><br>EXCI: 33.75 (19.73). Significant improvement from baseline to 1-month post (**p < 0.05**).<br><br>There were significant between-group differences (**p = 0.006**) between EXCITENS & EXCIEA, and between EXCIEA & EXCI (both in favour of EXCIEA).<br><br><u>Sorenson test:</u><br>EXCITENS: 30.44 (26.97). Change from baseline to 1-month post: p > 0.05.<br><br>EXCIEA: 74.0 (59.76). Significant improvement from baseline to 1-month post (**p < 0.05**).<br><br>EXCI: 47.0 (27.26). Significant improvement from baseline to 1-month post (**p < 0.05**).<br><br>There was a significant difference (**p = 0.01**) between EXCITENS & EXCIEA, in favour of EXCIEA.	NA

Dimer daLuz et al., 2019 [27]	1. CORE (n=10)<br>2. NMES (n=10)<br>3. COMB (n=10)	Static trunk endurance; back extensor endurance (Sorenson test)	Static trunk endurance:<br>CORE group improved from 30.4 (15.83) to 46.3 (19.67), **$p < 0.05$**.<br><br>NMES group changed from 29.9 (24.13) to 39.3 (25.29), $p > 0.05$.<br><br>COMB group improved from 53.30 (21.91) to 133.4 (53.02), **$p < 0.05$**.<br><br>There were significant differences (**$p < 0.05$**) between COMB & NMES, and COMB & CORE, both in favor of COMB.<br><br>Sorenson test:<br>CORE group improved from 35.0 (16.99) to 60.7 (15.74), **$p < 0.05$**.<br><br>NMES group improved from 35.1 (23.9) to 59.5 (28.3), **$p < 0.05$.**<br><br>COMB group improved from 46.6 (29.52) to 91.6 (23.77), **$p < 0.05$**.<br><br>There were significant differences (**$p < 0.05$**) between COMB & NMES, and COMB & CORE, both in favor of COMB.	NA	**At 6-months post:**<br>Static trunk endurance:<br>CORE: 37.1 (13.7).<br><br>NMES: 40.1 (23.24).<br><br>COMB: 83.6 (4.192). Significant improvement in COMB group from baseline to 6m-post (**$p < 0.05$**).<br><br>There were significant differences (**$p < 0.05$**) between COMB & NMES, and COMB and CORE, both in favor of COMB.<br><br>Sorenson test:<br>CORE: 58.1 (17.61). Significant improvement in CORE group from baseline to 6m-post (**$p < 0.05$**).<br><br>NMES: 52.1 (24.63).<br><br>COMB: 67.9 (22.78).<br><br>There were no significant between-group differences.
Kofotolis et al., 2008 [28]	1. RS (n=23)<br>2. TENS (n=23)<br>3. COMB (n=21)<br>4. PTENS (n=21)	Static flexion endurance (curl-up); static extension endurance (modified Sorenson back extension test); dynamic flexion endurance (curl-up); dynamic extension endurance (modified Sorenson back extension test);	Static flexion endurance:<br>RS group improved from 53.8 (4.3) to 71.4 (4.2), **$p < 0.05$.**<br><br>TENS group improved from 54.9 (3.5) to 59.2 (5.5), **$p < 0.05$.**	Static flexion endurance:<br>RS: 70.0 (11.6). Significant improvement from baseline to 1-month post-intervention (**$p < 0.05$**).<br><br>TENS: 58.2 (10.5).<br><br>COMB: 64.6 (19.7).	**At 2-months post:**<br>Static flexion endurance:<br>RS: 69.1 (14.9). Significant improvement from baseline to 2-months post-intervention (**$p < 0.05$**).<br><br>TENS: 58.8 (9.6).<br><br>COMB: 64.8 (16.2).

			COMB group improved from 57.1 (4.4) to 66.3 (5.3), **$p < 0.05$.**<br><br>PTENS group changed from 55.7 (5.1) to 53.4 (1.8), $p > 0.05$.<br><br>There were significant differences (**$p < 0.05$**) between RS & PTENS, RS & TENS, RS & COMB (all in favor of RS), COMB & PTENS, COMB & TENS (both in favor of COMB), TENS & PTENS (in favor of TENS).<br><br><u>Static extension endurance</u>: RS group improved from 80.5 (6.0) to 137.0 (6.9), **$p < 0.05$**.<br><br>TENS group changed from 81.1 (6.2) to 82.5 (6.2), $p > 0.05$.<br><br>COMB group improved from 80.6 (7.0) to 101.3 (9.3), **$p < 0.05$**.<br><br>PTENS group changed from 79.0 (9.3) to 79.0 (6.8), $p > 0.05$.<br><br>There were significant differences (**$p < 0.05$**) between RS & PTENS, RS & TENS, RS & COMB (all in favor of RS), COMB & PTENS, COMB & TENS (both in favor of COMB).<br><br><u>Dynamic flexion endurance</u>: RS group improved from 8.9 (1.9) to 12.1 (1.6), **$p < 0.05$**.	PTENS: 51.7 (11.5)<br><br>There were significant differences (**$p < 0.05$**) between RS & PTENS, RS & TENS (both in favor of RS), COMB & PTENS (in favor of COMB).<br><br><u>Static extension endurance</u>: RS: 139.8 (4.0). Significant improvement from baseline to 1-month post-intervention (**$p < 0.05$**).<br><br>TENS: 83.1 (4.3).<br><br>COMB: 107.3 (9.9). Significant improvement from baseline to 1-month post-intervention (**$p < 0.05$**).<br><br>PTENS: 78.6 (6.2).<br><br>There were significant differences (**$p < 0.05$**) between RS & PTENS, RS & TENS, RS & COMB (all in favor of RS), COMB & PTENS, COMB & TENS (both in favor of COMB).<br><br><u>Dynamic flexion endurance</u>: RS: 11.6 (2.1). Significant improvement from baseline to 1-month post-intervention (**$p < 0.05$**).<br><br>TENS: 7.8 (1.7).<br><br>COMB: 9.5 (1.3). Significant improvement from baseline to 1-month post-intervention (**$p < 0.05$**).<br><br>PTENS: 7.6 (1.2)	PTENS: 52.1 (8.5).<br><br>There were significant differences (**$p < 0.05$**) between RS & PTENS, RS & TENS (both in favor of RS), COMB & PTENS (in favor of COMB).<br><br><u>Static extension endurance</u>: RS: 140.3 (31.3). Significant improvement from baseline to 2-months post-intervention (**$p < 0.05$**).<br><br>TENS: 86.6 (32.1).<br><br>COMB: 111.2 (19.1). Significant improvement from baseline to 2-months post-intervention (**$p < 0.05$**).<br><br>PTENS: 78.3 (29.9)<br><br>There were significant differences (**$p < 0.05$**) between RS & PTENS, RS & TENS, RS & COMB (all in favor of RS), COMB & PTENS, COMB & TENS (both in favor of COMB).<br><br><u>Dynamic flexion endurance</u>: RS: 11.7 (5.1). Significant improvement from baseline to 2-months post-intervention (**$p < 0.05$**).<br><br>TENS: 8.0 (3.2).<br><br>COMB: 9.4 (2.2). Significant improvement from baseline to 2-months post-intervention (**$p < 0.05$**).<br><br>PTENS: 7.1 (3.0).

			TENS group changed from 7.8 (0.9) to 7.5 (0.8), $p > 0.05$.<br><br>COMB group improved from 7.8 (1.2) to 9.2 (1.3), $\mathbf{p < 0.05}$.<br><br>PTENS group changed from 7.8 (1.7) to 7.9 (1.2), $p > 0.05$.<br><br>There were significant differences ($\mathbf{p < 0.05}$) between RS & PTENS, RS & COMB, RS & TENS (all in favor of RS), COMB & PTENS, COMB & TENS (both in favor of COMB).<br><br><u>Dynamic extension endurance</u>:<br>RS group changed from 8.3 (1.6) to 11.4 (1.5), $p > 0.05$.<br><br>TENS group changed from 7.9 (1.1) to 8.8 (1.3), $p > 0.05$.<br><br>COMB group improved from 8.4 (1.2) to 9.5 (1.5), $\mathbf{p < 0.05}$.<br><br>PTENS group changed from 8.0 (0.8) to 8.2 (0.9), $p > 0.05$.<br><br>There were significant differences ($\mathbf{p < 0.05}$) between RS & PTENS, RS & TENS, RS & COMB (all in favor of RS), COMB & PTENS (in favor of COMB).	There were significant differences ($\mathbf{p < 0.05}$) between RS & PTENS, RS & COMB, RS & TENS (all in favor of RS), COMB & PTENS, COMB & TENS (both in favor of COMB).<br><br><u>Dynamic extension endurance</u>:<br>RS: 11.2 (1.3). Significant improvement from baseline to 1-month post-intervention ($\mathbf{p < 0.05}$).<br><br>TENS: 9.2 (1.2).<br><br>COMB: 10.0 (1.5). Significant improvement from baseline to 1-month post-intervention ($\mathbf{p < 0.05}$).<br><br>PTENS: 8.8 (1.2).<br><br>There were significant differences ($\mathbf{p < 0.05}$) between RS & PTENS, RS & TENS, RS & COMB (all in favor of RS), COMB & PTENS (in favor of COMB).	<u>Dynamic extension endurance</u>:<br>RS: 11.3 (1.2). Significant improvement from baseline to 2-month post-intervention ($\mathbf{p < 0.05}$).<br><br>TENS: 9.1 (1.3).<br><br>COMB: 9.7 (1.8). Significant improvement from baseline to 2-month post-intervention ($\mathbf{p < 0.05}$).<br><br>PTENS: 8.8 (1.2)<br><br>There were significant differences ($\mathbf{p < 0.05}$) between RS & PTENS, RS & TENS, RS & COMB (all in favor of RS), COMB & PTENS (in favor of COMB).
Pelegrini et al., 2019 [30]	1. AC (n=12)<br>2. C (n=12)	Trunk extensor endurance (horizontal board test)	AC group changed from 40.72 (14.18) to 48.38 (20.63), $p > 0.05$.	AC group: 40.12 (14.68).<br><br>C group: 28.19 (6.72).	NA

			C group changed from 38.46 (17.62) to 31.89 (8.81), p > 0.05<br>There was a significant between-group difference (**p = 0.0191, ES = -1.08**) in favour of the AC group.	There was a significant between-group difference (**p = 0.0176, ES = 1.08**) in favour of the AC group.	
Weissenfels et al., 2018 [17]	1. WB-EMS (n=15)<br>2. C (n=15)	Isometric trunk flexion strength; isometric trunk extension strength	<u>Maximum isometric trunk extension</u>:<br>WB-EMS group improved 7.26 (9.69) from baseline (**p < 0.01**).<br><br>CG regressed -1.03 (9.75) from baseline.<br><br>Between-group differences revealed a significantly greater improvement in isometric trunk extension strength in favour of WB-EMS group (**p = 0.038, ES = 0.853**)<br><br><u>Maximum isometric trunk flexion</u>:<br>WB-EMS group improved 6.79 (8.51) from baseline (**p < 0.01**).<br><br>CG improved 1.29 (8.62).<br><br>There was no significant between-group difference in score changes over time.	NA	NA
Weissenfels et al., 2019 [31]	1. WB-EMS (n-55)<br>2. CT (n=55)	Isometric trunk flexion strength (Back-Check 607); isometric trunk extension strength (Back-Check 607)	<u>Maximum isometric trunk extension</u>:<br>WB-EMS group improved 7.19 (8.82) from baseline (**p < 0.001**).<br><br>CT group improved 8.96 (8.78) from baseline.<br><br>There was no significant between-group difference in score changes over time.	NA	NA

			<u>Maximum isometric trunk flexion</u>:<br>WB-EMS group improved 7.30 (9.05) from baseline (**p < 0.001**).<br><br>CT group improved 6.61 (9.09) from baseline (**p < 0.001**).<br><br>There was no significant between-group difference in score changes over time.		

<u>LEGEND</u>: TENS = transcutaneous electrical nerve stimulation; NMES = neuromuscular electrical nerve stimulation; WB-EMS = whole-body electromyostimulation; STAB = stabilization exercise; SNMES = stabilization exercises + NMES; RC = Russian current; C = control; EXCI = exercise; EXCITENS = exercise + TENS; EXCIEA = exercise + electroacupuncture; CORE = core exercise; COMB = core exercise + NMES; RS = rhythmic stabilization; **COMB** = rhythmic stabilization + TENS: PTENS = placebo TENS; AC = Aussie current; CT = conventional training; MD = mean difference; ES = effect size

Supplementary Table S5 – Outcome: Paraspinal Muscle Thickness

**Study**	**Groups**	**Outcome/Tool**	**Result: post-intervention**	**Result: 1-month post**
Batistella et al., 2020 [32]	1. RC (n=11)<br>2. C (n=12)	Resting lumbar multifidus thickness (ultrasound)	RC group changed from 3.74 (0.45) to 3.92 (0.35), p > 0.05.<br><br>C group changed from 4.26 (0.77) to 4.07 (0.59), p > 0.05.<br><br>There was no significant between-group difference at post-intervention.	RC group: 3.80 (0.34).<br><br>C group: 3.97 (0.55).<br><br>There was no significant between-group difference at 1-month post-intervention.
Pelegrini et al., 2019 [30]	1. AC (n=12)<br>2. C (n=12)	Resting lumbar multifidus thickness (ultrasound)	AC group changed from 4.28 (0.59) to 4.51 (0.63), p > 0.05.<br><br>C group changed from 3.98 (0.57) to 3.79 (0.58), p > 0.05.<br><br>There was a significant between-group difference (**p = 0.0049, ES = 1.17**) in favour of the AC group.	AC group: 4.23 (0.6).<br><br>C group: 3.71 (0.50).<br><br>There was a significant between-group difference (**p = 0.0161, ES = 0.91**) in favour of the AC group.

<u>LEGEND</u>: RC = Russian current; C = control; AC = Aussie current; ES = effect size
